# Disease diagnosis in primary care in Uganda

**DOI:** 10.1186/1471-2296-15-165

**Published:** 2014-10-08

**Authors:** Martin Kayitale Mbonye, Sarah M Burnett, Robert Colebunders, Sarah Naikoba, Jean-Pierre Van Geertruyden, Marcia R Weaver, Allan Ronald

**Affiliations:** Infectious Diseases Institute, Makerere University, Mulago Hospital Complex, P.O. BOX 22418, Kampala, Uganda; Accordia Global Health Foundation, Washington, DC USA; Department of Epidemiology and Social Medicine, University of Antwerp, Campus Drie Eiken, S.4.10 Universiteitsplein 1, B-2610 Wilrijk, Antwerp, Belgium; Department of Clinical Sciences, Institute of Tropical Medicine, Antwerp, Belgium; International Training and Education Center for Health (I-TECH), Department of Global Health, University of Washington, Seattle, USA; Department of Medicine, University of Manitoba, Winnipeg, Manitoba Canada

**Keywords:** Primary care, Disease diagnosis, Uganda

## Abstract

**Background:**

The overall burden of disease (BOD) especially for infectious diseases is higher in Sub-Saharan Africa than other regions of the world. Existing data collected through the Health Management Information System (HMIS) may not be optimal to measure BOD. The Infectious Diseases Capacity Building Evaluation (IDCAP) cooperated with the Ugandan Ministry of Health to improve the quality of HMIS data. We describe diagnoses with associated clinical assessments and laboratory investigations of outpatients attending primary care in Uganda.

**Methods:**

IDCAP supported HMIS data collection at 36 health center IVs in Uganda for five months (November 2009 to March 2010) prior to implementation of the IDCAP interventions. Descriptive analyses were performed on a cross-sectional dataset of 209,734 outpatient visits during this period.

**Results:**

Over 500 illnesses were diagnosed. Infectious diseases accounted for 76.3% of these and over 30% of visits resulted in multiple diagnoses. Malaria (48.3%), cough/cold (19.4%), and intestinal worms (6.6%) were the most frequently diagnosed illnesses. Body weight was recorded for 36.8% of patients and less than 10% had other clinical assessments recorded. Malaria smears (64.2%) and HIV tests (12.2%) accounted for the majority of 84,638 laboratory tests ordered. Fewer than 30% of patients for whom a laboratory investigation was available to confirm the clinical impression had the specific test performed.

**Conclusions:**

We observed a broad range of diagnoses, a high percentage of multiple diagnoses including true co-morbidities, and underutilization of laboratory support. This emphasizes the complexity of illnesses to be addressed by primary healthcare workers. An improved HMIS collecting timely, quality data is needed. This would adequately describe the burden of disease and processes of care at primary care level, enable appropriate national guidelines, programs and policies and improve accountability for the quality of care.

**Electronic supplementary material:**

The online version of this article (doi:10.1186/1471-2296-15-165) contains supplementary material, which is available to authorized users.

## Background

Despite recent reductions in both morbidity and mortality, the burden of diseases (BOD) in Sub-Saharan Africa (SSA) remains high when compared with the rest of the world, particularly due to the high burden of infectious diseases [1]. While malaria, tuberculosis and HIV/AIDS remain the leading causes of illness and death in the region, maternal and neonatal complications, diarrheal diseases, pneumonia, and malnutrition are drivers of ‘health loss’, particularly in children [1]. Concurrently, there has also been an increase in BOD due to non-communicable diseases (NCD) in adults [1–7].

Little is know about the spectrum of clinical diagnoses encountered at primary care facilities in many SSA countries, including Uganda. Organizations such as the World Bank, the Institute for Health Metrics and Evaluation [1], the World Health Organization [7], and others [2–5] utilize routine health information systems, alongside national representative household surveys and health and demographic surveillance sites, for assessing, estimating and documenting global, regional, country and disease specific BOD [8]. Well collected and analyzed data inform clinical decision support systems [9, 10], and enable assessment of disease risk, setting of priorities, evaluations of cost-effectiveness [11] and program efficiency [12], and national planning and policy formulation [13].

In Uganda, health information data are collected through the Health Management Information System (HMIS) [14, 15] at all levels of the health system. The Medical Form Five (MF5) acts as the case record form and is used to document individual patient data during visits to the outpatient department. Data collected using the MF5 are transcribed into the patient registers, which stay at the health facilities while the original MF5 is retained by the patient [15]. These notes guide patient management during subsequent visits. On a monthly basis, data in the patient registers are aggregated and forwarded to the district and the Ministry of Health (MoH). The Uganda MoH Resource Centre has the overall responsibility for processing national data collected through the HMIS and use these data to compile annual health sector performance reports [16] statistical abstracts [17] and policy formulation.

Although HMIS data could be a powerful resource for estimating the BOD and developing clinical decision support systems [18, 19], its use has been limited by quality limitations, such as underreporting, data loss during aggregation, and erroneous diagnoses [20–25].

The Integrated Infectious Disease Capacity Building Evaluation (IDCAP) sought to estimate the effect of a training program followed by on-site support (OSS) [26] on individual clinical competence [27] and practice, facility-level performance [28, 29], and population-based mortality of children under five. To measure facility performance, the IDCAP evaluation team cooperated with the Uganda MoH to improve the quality of HMIS data through provision of data entry staff and equipment, training and support of supervision visits [30].

We present a review of outpatient visit records from primary care facilities in Uganda from November 2009 to March 2010. While these visits were primarily intended to serve as a baseline for the IDCAP training and quality improvement interventions, they provide a patient-level dataset to examine the potential of using a strengthened HMIS to estimate the BOD.

This article describes outpatient demographic characteristics, common symptoms, investigations, diagnoses and multiple diagnoses recorded in the MF5. It also demonstrates the limitations of clinical diagnosis.

## Methods

### Study objectives

To describe diagnoses, multiple diagnoses or comorbidities, clinical assessments and the extent to which diagnoses are confirmed by laboratory investigations among outpatients presenting at primary care facilities in rural Uganda.

### Study design

This is a cross sectional study that analyzes baseline data collected prior to a large mixed design intervention study described in Naikoba et al. [30]. After these baseline data were collected, two IDCAP interventions were implemented 1) the Integrated Management of Infectious Diseases (IMID) course and 2) on-site support. IMID was for two selected mid-level practitioners (mainly clinical officers and registered nurses) from each of the 36 health facilities conducted at the Infectious Diseases Institute, Makerere University (IDI). It involved a three-week classroom based core course, followed by two one-week booster courses at 12 and 24 weeks after the core course as described in Miceli et al. [26]. On-site support was training and continuous quality improvement which took place at a random sample of 18 of the 36 health facilities. All health workers were invited to participate in the on-site support training as described in Naikoba et al. [30] and Miceli et al. [26].

### Study sites and participants

**Figure 1:**
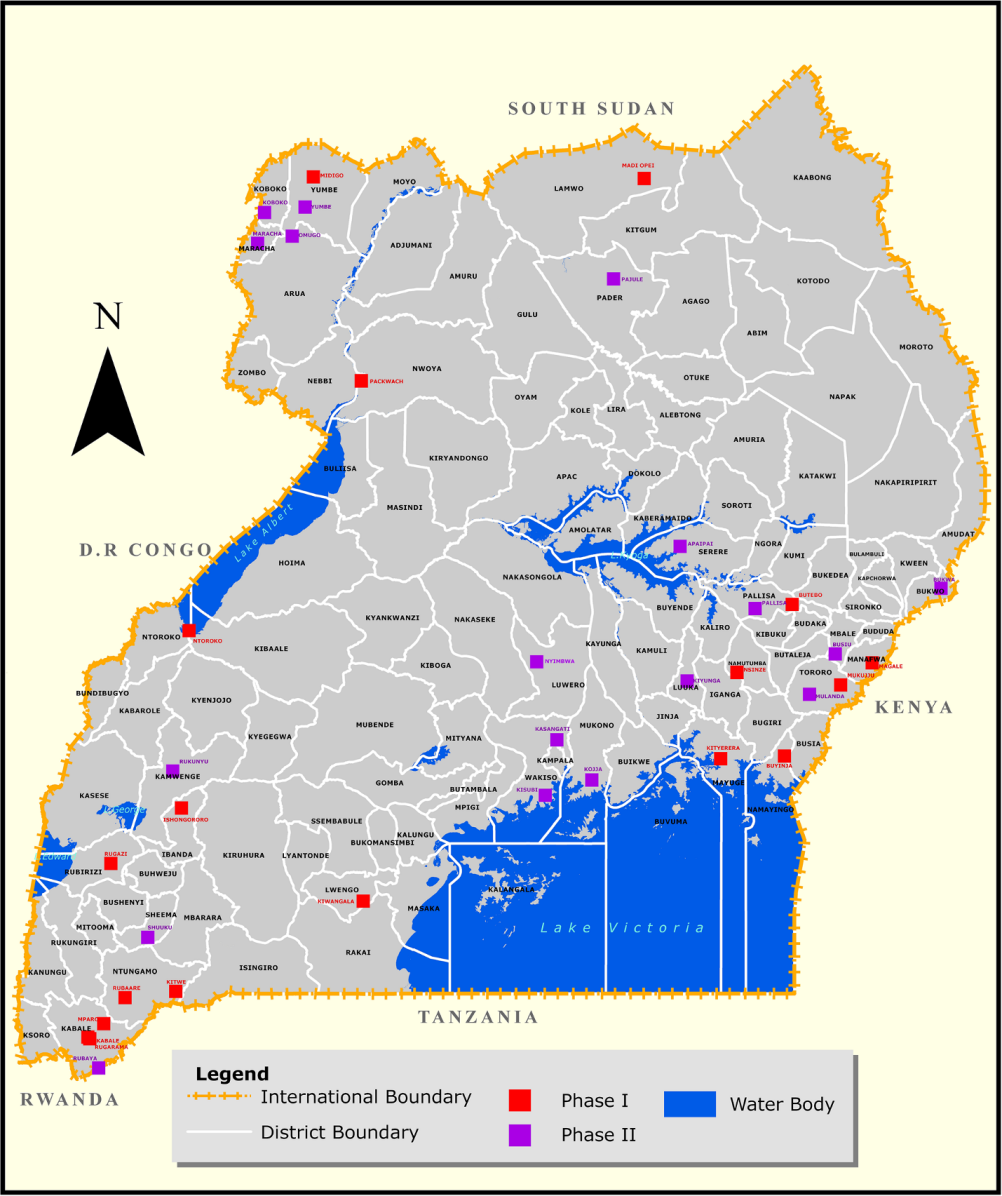
**Map of Uganda showing location of IDCAP sites.** It is a map of Uganda showing location of all the health facilities that participated in the IDCAP study.

IDCAP was implemented at 36 health center IVs (HCIV) or comparable health facilities from all administrative regions of Uganda, as depicted in a map in Figure 1, that met the inclusion criteria [30]. The government of Uganda managed thirty sites, five were private not-for-profit sites and one was a private for-profit site with government subsidies. Five sites are small hospitals while 31 are HCIVs. All are the highest healthcare referral point for a health sub-district [31, 32]. Each is expected to serve a population of about 100,000 people in the health sub-district, providing basic preventive, curative and referral services with limited inpatient wards. HCIVs also conduct some emergency and surgical and obstetric procedures [17, 32]. Staffing norms and level of staffing for a HCIV can be obtained from the 2010 MoH Human Resources for Health Audit Report [33]. Participants included all outpatients at these health facilities.

### Data collection, entry and validation

**Figure 2:**
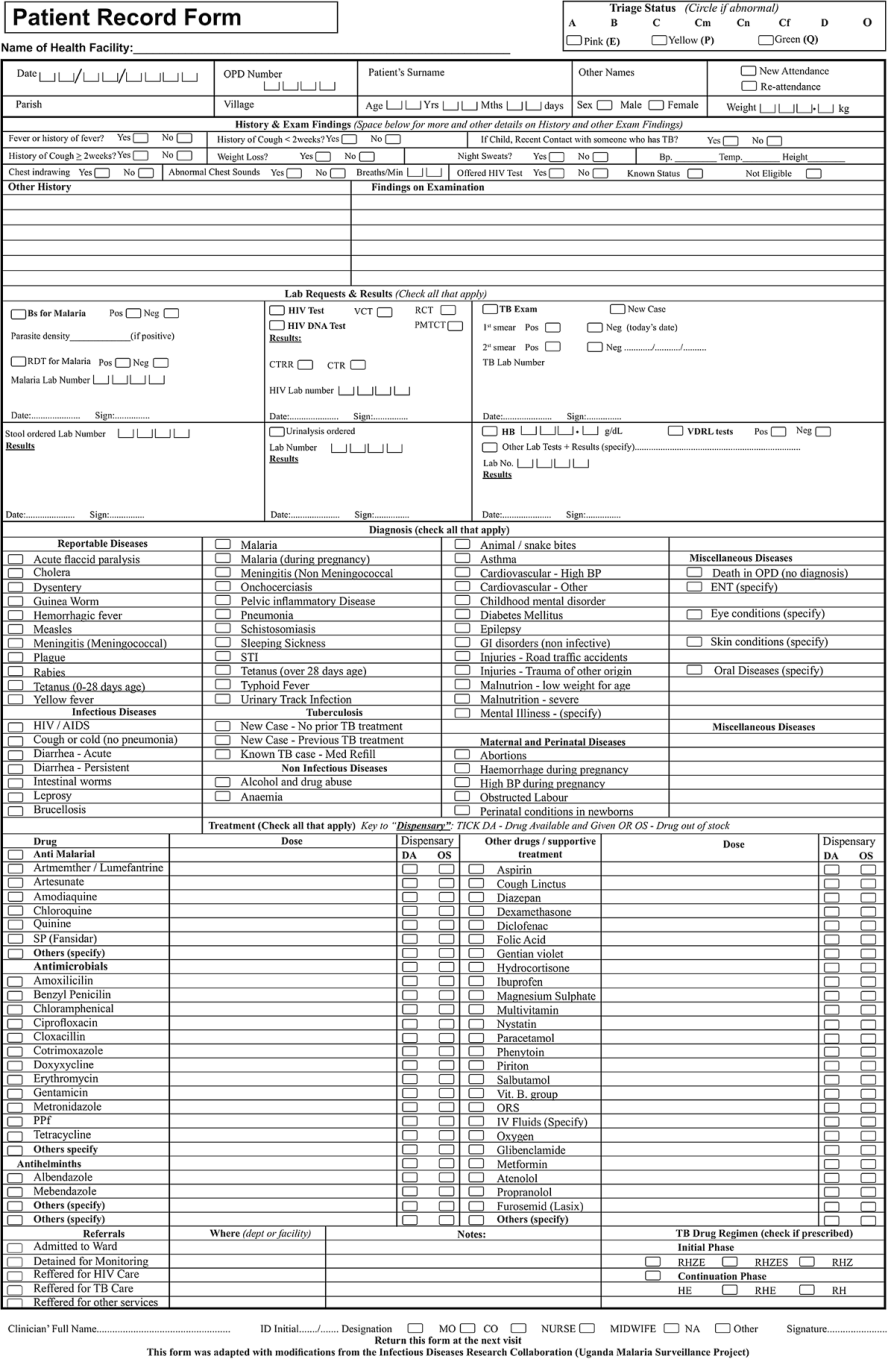
**The Medical Form 5 used during IDCAP data collection.** It is the revised Uganda Ministry of Health Outpatient Medical Form used to capture patient’s data during routine care.

Data were collected using the MF5 depicted in Figure 2, which was initially modified by the Uganda Malaria Surveillance Project [12] and further revised by IDCAP [28], and includes links between clinical and laboratory data, and ‘tick boxes’ for history, laboratory investigations, diagnoses and drug prescriptions.

Prior to collecting data, a team comprised of a data management specialist, a medical officer, a clinical officer and a nursing officer introduced the MF5 at each health facility during a one-day training for all health workers. Two key training activities were: 1) an explanation of all variables on the MF5 and the process of collecting outpatient data, and 2) a practical session with the form during routine care. At the end of the day, the health workers met with the trainers to share experiences and offer suggestions to optimize use of the MF5 within the conditions unique to each health facility. Four training teams were created with each team supporting nine health facilities.

All health facilities consistently started using the MF5 on November 1, 2009. The MF5 collected patient data at various points of care; data on triage and patient demographics at the reception, data on illness history, diagnosis, treatment and referral from the clinician, data on diagnostic investigations and results from laboratory personnel and data on drug availability from a dispenser or pharmacist.

Data were entered into EpiInfo 3.2^®^ (Centers for Disease Control and Prevention, Atlanta GA, US) and transmitted to the central database at the IDI for aggregation, cleaning and analysis. A team of laboratory technicians and clinicians at the IDI supported data cleaning and coding of diagnoses guided by the ICD-10. The process of data collection, entry and validation has been explained previously in more detail [28].

### Statistical analysis

Frequencies and percentages were used for most analyses while cross tabulations were performed to establish relationships between different variables of interest. Although 238,248 patient visits occurred during the data collection period, 28,514 (12%) MF5s with no record of age or sex variables were excluded, leaving 209,734 records for analysis. All statistical analyses were performed using Stata version 11.0 (StataCorp LP, College Station TX, 2009), and Microsoft Office Excel 2007 (Microsoft Corporation, Redmond WA, 2007).

### Ethics Statement

The original IDCAP protocol was reviewed and approved by the School of Medicine Research and Ethics Committee of Makerere University and the Uganda National Council for Science and Technology. Data collected for the HMIS were part of routine reporting at health facilities and did not require informed consent. The University of Washington Human Subjects Division determined that IDCAP did not meet the regulatory definition of research under 45 CFR 46.102(d). This secondary analysis of anonymous data was also exempt from review by the University of Antwerp ethical review board. Anonymous data are available for public use and instructions for requesting them are on the Accordia Global Health Foundation website (http://www.accordiafoundation.org/IDCAP/data).

## Results

Demographic characteristics, symptoms and exams performed and a summary of diagnosis and treatment decisions for 209,734 patient visits with acute presentations relating to illness or injury are summarized in Table 1. Of these, 181,529 (97.1%) were initial presentations for unscheduled appointments. While male and female infants and children under five were equally likely to visit the HCIVs (30,311 vs. 30,381 visits), there were 46.9% more visits for females aged 14–49 years (85,138 vs. 45,177 males). Triage status according to the World Health Organization (WHO) Emergency Triage, Assessment and Treatment (ETAT) guidelines [34] were recorded for only 67,997 (32.4%) visits, of which 6,936 (10.2%) had ‘danger signs’ requiring immediate assessment and treatment.

**Table 1:** **Characteristics of outpatients**

Parameters	Male (N = 82,626)	Female (N = 127,108)	Total (N = 209,734)
	N (%)	N (%)	N (%)
**Age**			
< 1 month (Neonates)	336 (0.4)	364 (0.3)	700 (0.3)
1 – 11 months	8,996 (10.9)	9,018 (7.1)	18,014 (8.6)
1 – 4 years	20,979 (25.4)	20,999 (16.5)	41,978 (20.0)
5 – 13 years	15,568 (18.8)	20,004 (15.7)	35,572 (17.0)
14 – 49 years	29,609 (35.8)	65, 134 (51.2)	94,743 (45.2)
50+	7,138 (8.6)	11,589 (9.1)	18,727 (8.9)
**Total attendance**			
New visits	70,973 (85.9)	110,556 (87.0)	181,529 (86.6)
Repeat visits	2,310 (2.8)	3,051 (2.4)	5,361 (2.6)
Missing data	9,343 (11.3)	13,501 (10.6)	22,844 (10.9)
**Triage status recorded** ^**‡**^			
Standard	19,178 (23.2)	30,482 (24.0)	49,660 (23.7)
Priority	4,542 (5.5)	6,859 (5.4)	11,401 (5.4)
Emergency	2,909 (3.5)	4,027 (3.2)	6,936 (3.3)
Missing data (no triage data recorded)	55,997 (67.8)	85,740 (67.4)	141,737 (67.6)
**Symptoms recorded**			
Fever	37,254 (45.1)	52,342 (41.2)	89,596 (42.7)
No fever	5,164 (6.2)	8,886 (7.0)	14,050 (6.7)
Data not recorded on fever	40,210 (48.7)	65,880 (51.7)	106,088 (50.6)
Cough	22,890 (27.7)	31,961 (25.1)	54,851 (26.2)
Cough < 2 weeks	19,792 (24.0)	27,589 (21.7)	47,381 (22.6)
Cough > =2 weeks	2,908 (3.5)	4,121 (3.2)	7,029 (3.4)
Cough (not duration not determined)	190 (0.2)	251 (0.2)	441 (0.2)
No cough	8,857 (10.7)	14,297 (11.2)	23,144 (11.0)
Data not recorded on cough	50,881 (61.6)	80,850 (63.6)	131,739 (62.8)
Abnormal chest sounds among patients with a cough	88 (0.3)	104 (0.3)	192 (0.4)
No abnormal chest sounds	764 (3.3)	1,087 (3.4)	1,851 (3.4)
No data on abnormal chest sounds	22,038 (96.3)	30,770 (96.3)	52,808 (96.3)
Breaths per minute among patients with a cough	51 (0.2)	50 (0.2)	101 (0.2)
No data on breaths per minute	22,839 (99.8)	31,911 (99.8)	54,750 (99.8)
Chest in drawing among children under five with a cough	67 (0.5)	70 (0.6)	137 (0.5)
No chest in drawing	364 (2.9)	368 (2.9)	732 (2.9)
Missing data on chest in drawing	12,023 (96.5)	12,068 (96.5)	24,091 (96.5)
Night sweats among patients with cough > =2 weeks	152 (5.2%)	147 (3.6)	299 (4.3)
No night sweats	372 (12.8)	560 (13.6)	932 (13.3)
No data on night sweats	2,384 (82.0)	3,414 (82.8)	5,798 (82.5)
Weight loss among patients with cough > =2 weeks	138 (4.7)	114 (2.8)	252 (3.6)
No weight loss	446 (15.3)	651 (15.8)	1,097 (15.6)
No data on weight loss	2,324 (79.9)	3,356 (81.4)	5,680 (80.8)
Child under 14 with a cough > =2 weeks in contact with a TB case	37 (0.22)	43 (0.23)	80 (0.23)
Child not in contact with a TB case	2,802 (16.6)	3,920 (21.1)	6,722 (19.0)
No data on child contact with a TB case	14,005 (83.2)	14,783 (78.7)	28,618 (81.0)
**Exams recorded**			
Temperature	5,688 (6.9)	8,132 (6.4)	13,820 (6.6)
Weight	30,980 (37.5)	46,161 (36.3)	77,141 (36.8)
Blood Pressure	522 (0.63)	1,247 (0.98)	1,769 (0.84)
Height	21 (0.03)	29 (0.02)	50 (0.02)
**No symptom and exam checkboxes filled**	33,915 (41.1)	55,009 (43.3)	88,924 (42.4)
**Diagnosis and treatment decisions**			
Diagnosis recorded	70,721 (85.6)	109,771 (86.4)	180,492 (86.1)
Treatment prescribed	79,639 (96.4)	123,554 (97.2)	203,193 (96.9)
Referred for further care	1,712 (2.1)	2,251 (1.8)	3,963 (1.9)
Detained for monitoring	127 (0.16)	170 (0.13)	297 (0.14)
Admitted	5,086 (6.2)	5,657 (4.5)	10,743 (5.1)

**‡**Triage status was established according to the World Health Organization Emergency Triage, Assessment and Treatment (ETAT) guidelines.

Few patients had their clinical exams recorded: Body weight (77,141; 36.8%), temperature (13,820; 6.6%) height (50; 0.024%) and blood pressure (BP) (1,769; 0.85%). BP recording was often done for older patients (3.9% among patients 50 or more years) compared to younger patients (1.1% among patients 14–49 years and 0.04% among patients less than five years).

The most frequently reported symptoms were fever, which was recorded in 89,596 (42.7%) visits, of which only 2,041 (2.3%) were confirmed by a thermometer (>37.5°C) and cough which was recorded in 54,851 (26.1%) visits, of which 7,029 (12.8%) was prolonged for two or more weeks.

Among three assessments for pneumonia recorded in the MF5, breathing rate and abnormal chest sounds were respectively assessed in 101 (0.18%) and 2,043 (3.7%) of 54,851 patients with a cough, while chest in-drawing was assessed in 869 (3.5%) of 24,960 children less than five years with a cough.

Among the three assessments for tuberculosis, night sweats and weight loss were respectively assessed in 17.5% and 19.2% of 7,029 patients with a cough for two or more weeks. Child contact with a TB case was assessed in 474 (11.1%) of 4,257 children less than 14 years with a cough for two or more weeks.

Overall, 180,492 (86.1%) visits had at least one diagnosis identified on the MF5, 203,193 (96.9%) received a drug prescription, 10,743 (5.1%) were admitted for further care, 3,963 (1.9%) were referred elsewhere for further care and 297 (0.14%) were detained for a short time for close monitoring.

### Diagnosis

Overall, over 500 distinct diagnoses (see Additional file 1) were made. Infectious diseases (76.3%) were the commonly diagnosed illnesses, followed by NCD (10.9%) and diagnoses that could not be determined according to the ICD-10 (10.6%) (Table 2). Illnesses that could not be determined according to the ICD-10 included: fatigue, vomiting and nausea, dizziness, and unspecified pain among others.

**Table 2:** **Summary of disease episodes by category of disease diagnosed**

Disease category	N (%), n = 254,983
Infectious/communicable diseases	194,492 (76.3)
Non communicable diseases & injuries	27,891 (10.9)
Diagnosis not determined according to the ICD-10	27,081 (10.6)
Indeterminate/ineligible diagnosis (diagnosis was written on the MF5 but could not be understood)	3,348 (1.3)
Diseases of epidemic potential	1,189 (0.5)
Maternal and perinatal conditions	760 (0.3)
Neglected tropical diseases	222 (0.1)

Similarly, for any specific diagnosis that required a laboratory investigation, fewer than 30% of patients were confirmed by a diagnostic test (Table 3). For example, only 17.8% of 101,266 malaria diagnoses were confirmed.

**Table 3:** **Distribution of commonly diagnosed diseases**

No	Diagnosis	National (MoH – HMIS reported)†	N = 209,734		Median age	Sex Ratio F/M	Laboratory confirmed N (%)	Multiple diagnoses** N (%)
			N	% (95% CI)				
**Top 20 diagnoses**								
1	Malaria	47.3	101,266	48.3 (48.1, 48.5)	10	1.47	18,051 (17.8)	48,954 (48.3)
2	Cough or cold	23.7	40,669	19.4 (19.2, 19.6)	9	1.48	N/A	27,295 (67.1)
3	Intestinal worms	6.4	13,892	6.6 (6.5, 6.7)	13	1.61	198 (1.4)	10,525 (75.8)
4	GI disorders*	2.8	11,519	5.5 (5.4, 5.6)	30	2.32	N/A	6,347 (55.1)
5	Pneumonia	3.2	8,335	3.9 (3.9, 4.1)	3	1.21	N/A	6,003 (72.0)
6	Diarrhea	3.6	8,137	3.9 (3.8, 4.0)	1	1.15	N/A	6,409 (78.8)
7	Skin diseases	3.7	8,028	3.8 (3.7, 3.9)	12	1.26	N/A	3,689 (46.0)
8	Urinary Tract Infection	2.3	7,043	3.4 (3.3, 3.4)	27	1.99	500 (7.1)	3,886 (55.2)
9	Oral diseases	_	4,832	2.3 (2.2, 2.4)	21	1.48	N/A	1,146 (23.7)
10	Injuries, Trauma*	_	4,618	2.2 (2.1, 2.3)	22	0.90	N/A	1,095 (23.7)
11	Eye conditions	2.4	3,782	1.8 (1.7, 1.9)	16	1.20	N/A	1,736 (45.9)
12	Ear Nose & Throat Conditions	_	3,762	1.8 (1.7, 1.9)	17	1.62	N/A	1,787 (47.5)
13	Pelvic Inflammatory Disease	_	3,486	1.7 (1.6, 1.7)	30	N/A	N/A	1,790 (51.4)
14	Sexually Transmitted Infections	2.8	3,338	1.6 (1.5, 1.7)	28	2.19	117 (3.5)	1,491 (44.6)
15	Anemia*	_	1,515	0.72 (0.69, 0.76)	2	1.19	408 (27.0)	1,307 (86.3)
16	Cardiovascular – High Blood Pressure*	_	1,437	0.69 (0.65, 0.73)	55	2.76	N/A	748 (52.1)
17	Typhoid fever	_	1,390	0.66 (0.63, 0.70)	25	1.52	302 (21.7)	829 (59.6)
18	HIV/AIDS	_	1,275	0.61 (0.58, 0.64)	32	1.82	251 (19.7)	720 (56.5)
19	Asthma*	_	1,095	0.52 (0.49, 0.55)	36	1.58	N/A	475 (43.4)
20	Arthritis, all cases	_	1,074	0.51 (0.48, 0.54)	46	2.43	N/A	453 (42.2)
**Top 5 diseases of epidemic potential**								
1	Dysentery	0.37	915	0.44 (0.41, 0.47)	14	1.24	41 (4.5)	558 (61.0)
2	Acute Flaccid paralysis	0.00	77	0.04 (0.03, 0.05)	15	2.67	N/A	60 (77.9)
3	Meningitis (Meningococcal)	0.00	66	0.03 (0.02, 0.04)	13.5	0.89	0 (0)	23 (34.9)
4	Measles	0.00	45	0.02 (0.015, 0.028)	2	0.8	N/A	33 (73.3)
5	Yellow Fever	0.00	17	0.01 (0.004, 0.012)	16	1.83	N/A	9 (52.9)
**Top 5 neglected tropical diseases**								
1	Schistosomiasis	_	78	0.04 (0.03, 0.04)	18.5	1.33	4 (5.1)	58 (74.4)
2	Leprosy	_	45	0.021 (0.02, 0.03)	40	1.00	0 (0)	32 (71.1)
3	Filiarisis	_	44	0.021 (0.01, 0.03)	40	2.39	0 (0)	27 (61.4)
4	Onchocerciasis	_	31	0.015 (0.01, 0.02)	25	2.88	1 (3.2)	17 (54.8)
5	Guinea worms	_	19	0.009 (0.001, 0.01)	11	1.71	N/A	13 (68.2)
**Top 5 maternal and perinatal conditions‡**								
1	Abortion	_	298	0.14 (0.13, 0.16)	24.0	N/A	N/A	89 (29.9)
2	Perinatal conditions in newborns	_	81	0.04 (0.03, 0.05)	0.00	0.98	N/A	18 (22.2)
3	Puerperal sepsis	_	43	0.02 (0.01, 0.02)	25.0	N/A	N/A	16 (37.2)
4	Pregnancy	_	43	0.02 (0.01, 0.02)	22.0	N/A	3 (7)	13 (30.2)
5	Ovarian cyst	_	35	0.02 (0.01, 0.02)	30.0	N/A	N/A	18 (51.4)

*Top 5 Non-Communicable Diseases.

**Percent is the proportion of patients with any given diagnosis who also had at least one other diagnosis.

†National MOH statistics are not available for all diagnoses.

**‡**Data only represents maternal and perinatal conditions that presented in the outpatient department; most patients with these conditions present at the specialized maternal and child health clinics including, antenatal, maternity and postnatal clinics.

For diagnoses where national statistics were available, our sample of 36 HCIVs had comparable prevalence (Table 3). Malaria (48.3%), cough/cold (19.4%), and intestinal worms (6.6%) were the commonly diagnosed illnesses.

For each of the top 20 commonly diagnosed illnesses, more females were diagnosed with illnesses than males, with exception of injuries and trauma (Female to Male ratio: 0.9). In some instances, female diagnoses were more than two times higher than that of males (sexually transmitted infections, gastrointestinal (GI) disorders, high BP and arthritis).

Many of the diagnoses were evenly distributed across age groups with some exceptions (see Additional file 2). Malaria (median age = 10 years), diarrhea (median age = 1 year), pneumonia (median age = 3 years) and anemia (median age = 2 years) were often diagnosed among younger patients, while arthritis (median age = 44 years) and high BP (median age = 55 years) were often diagnosed among older patients.

Several cases of diseases of epidemic potential were reported with dysentery (915, 0.44%) the most frequently diagnosed. Acute flaccid paralysis was diagnosed in 77 patients; meningococcal meningitis in 66; measles in 45 and yellow fever in 17. The top five most prevalent neglected tropical diseases included 78 cases of schistosomiasis; 45 of leprosy; 44 of filariasis (general); 31 of onchocerciasis and 19 of guinea worm.

The top 5 NCD appeared among the top 20 commonly diagnosed illnesses with the commonest being: miscellaneous GI disorders 11,519 (5.5%), injuries/trauma 4,618 (2.2%), anemia 1,515 (0.7%), high BP 1,437 (0.7%) and arthritis 1,074 (0.5%).

### Laboratory investigations

Table 4 shows the distribution of commonly conducted laboratory tests, results, and link between positive/abnormal results and diagnosis for related illnesses.

**Table 4:** **Laboratory investigations conducted: top 15 laboratory tests**

Rank	Laboratory test	Total tests	Results received	Positive or abnormal test result	Diagnosed with a related illness
		N (%)	N (%)	N (%)	N (%)
1	Malaria Test (Blood Smear or RDT)	54,344 (64.2)	48,280 (88.8)	22,806 (47.2)	18,051 (79.1)
2	HIV Test (Rapid Test or DNA PCR)	10,301 (12.2)	8,768 (85.1)	844 (9.6)	251 (29.7)
3	Urinalysis	4,672 (5.5)	3,125 (66.9)	1,349 (43.2)	702 (52.0)
4	Stool Analysis	3,713 (4.4)	2,252 (60.7)	648 (28.8)	414 (63.9)
5	Hemoglobin (Hb)	3,467 (4.1)	2,608 (75.2)	2,071 (79.4)	408 (19.7)
6	VDRL (Venereal Disease Research Lab test)	2,361 (2.8)	1,812 (76.7)	300 (16.6)	31 (10.3)
7	Widal	2,273 (2.7)	1,565 (68.9)	480 (30.7)	302 (63.0)
8	TB AFB Smear	1,347 (1.6)	757 (56.2)	113 (14.9)	60 (53.1)
9	BAT (Brucella Agglutination Test)	808 (1.0)	569 (70.4)	59 (10.4)	30 (50.9)
10	Pregnancy Test	306 (0.4)	258 (84.3)	107 (41.5)	3 (2.8)
11	Random Blood Sugar	234 (0.3)	176 (75.2)	50 (28.4)	25 (50.0)
12	Blood Grouping and/or Blood Cross March	225 (0.3)	188 (73.7)	N/A	N/A
13	RPR (Rapid Plasma Reagin Test)	80 (0.1)	57 (71.3)	7 (12.2)	2 (28.6)
14	CATT (Card agglutination test for trypanasomiasis)	81 (0.1)	74 (91.4)	5 (6.8)	0 (0)
15	Sickle cell test	64 (0.1)	32 (50.0)	5 (15.6)	1 (20.0)

Overall, 84,638 laboratory tests were ordered during 56,117 (28.8%) visits. Not all tests done had results recorded (range: 50% for sickle cell tests and 91.4% for CATT). Of the 48,280 malaria tests with results, 22,806 (47.2%) were positive for malaria. Although malaria (48.5%) topped the disease prevalence list, only 17.8% was confirmed (Table 3). While 10,321 patients were offered an HIV test, the test was not conducted for 20 of them. Of the 10,301 HIV tests conducted, 8,768 (85.1%) had a result recorded, of which 844 (9.6%) were positive. Two tests (VDRL and RPR) to determine syphilis and other STIs were done of which 16.6% and 12.2% respectively were positive. For urinalysis and stool analysis, 43.2% and 28.8% of the tests done indicated a positive result, while 79.4% of 2,608 hemoglobin tests with results indicated anemia.

Usually, patients who test positive for malaria should be assessed for anemia. Additional analysis showed that, among the 22,806 patients with a positive smear result for malaria, only 1,438 (6.3%) had hemoglobin measured of which 1,188 (82.6%) were anemic.

Generally, apart from malaria (79.1%), a lot of positive/abnormal test results did not have a record of diagnosis for a related illness.

### Multiple diagnoses and comorbidities

Among the 180,492 (86.1%) outpatient visits with diagnoses, 63,414 (35.1%) had multiple diagnoses (Table 3). For example, of the 101,266 malaria diagnoses, 48,954 (48.3%) had additional diagnoses. Table 5 reports comorbidities among the 10 most commonly diagnosed illnesses. The bold text across the diagonal of the table presents mono diagnoses. Column and row percentages are presented in the upper and lower parts of the diagonal of the table respectively. For example, in the upper diagonal (column percentages) of the table, 22,217 (55.6%) of the 40,669 cough or cold cases were also diagnosed with malaria while in the lower diagonal, 22,217 (21.9%) of the 101,266 malaria cases were also diagnosed with cough or cold. Among the top 10 diagnoses reported in Table 5, there was a disproportionate overlap among malaria, cough/cold, pneumonia and diarrhea. In 8,335 patients diagnosed with pneumonia and 8,137 with diarrhea, 5,302 (63.6%) and 4,822 (59.3%) respectively were also diagnosed with malaria. Similarly, a high proportion of patients with diarrhea were also diagnosed with cough/cold (23.1%). Of those diagnosed with intestinal worms or urinary tract infections, there was a high percentage also diagnosed with GI problems (6.1% and 5.6%, respectively).

**Table 5:** **Multiple diagnoses among each of the top 10 diagnoses**

N (Column %)	Malaria	Cough or cold	Intestinal worms	Gastro Intestinal Disorders	Pneumonia	Diarrhea	Skin diseases	Urinary tract infections	Oral diseases	Injuries/Trauma	Overall total
N (Row %)											
Malaria	**52,312 (51.7%)**	22,217 (54.6%)	6,903 (49.7%)	3,200 (27.8%)	5,302 (63.6%)	4,822 (59.3%)	1,866 (23.2%)	2,043 (29.0%)	677 (14.0%)	426 (10.2%)	**101,266**
Cough or cold	22,217 (21.9%)	**13,374 (32.9%)**	2,559 (18.4%)	1,180 (10.2%)	0 (0.0%)	1,877 (23.1%)	794 (9.9%)	369 (5.2%)	286 (5.9%)	191 (4.6%)	**40,669**
Intestinal worms	6,903 (6.8%)	2,559 (6.3%)	**3,367 (24.2%)**	845 (7.3%)	465 (5.6%)	657 (8.1%)	562 (7.0%)	505 (7.2%)	71 (1.5%)	72 (1.7%)	**13,892**
Gastro Intestinal disorders	3,200 (3.2%)	1,180 (2.9%)	845 (6.1%)	**5,172 (44.9%)**	153 (1.8%)	191 (2.3%)	207 (2.6%)	394 (5.6%)	58 (1.2%)	71 (1.7%)	**11,519**
Pneumonia	5,302 (5.2%)	0 (0.0%)	465 (3.3%)	153 (1.3%)	**2,332 (28.0%)**	349 (4.3%)	113 (1.4%)	82 (1.2%)	53 (1.1%)	19 (0.5%)	**8,335**
Diarrhea	4,822 (4.8%)	1,877 (4.6%)	657 (4.7%)	191 (1.7%)	349 (4.2%)	**1,728 (21.2%)**	134 (1.7%)	79 (1.1%)	92 (1.9%)	18 (0.4%)	**8,137**
Skin diseases	2,049 (2.0%)	794 (2.0%)	562 (4.0%)	207 (1.8%)	113 (1.4%)	134 (1.6%)	**4,339 (54.0%)**	125 (1.8%)	53 (1.1%)	85 (2.0%)	**8,028**
Urinary tract infections	2,043 (2.0%)	369 (0.9%)	505 (3.6%)	394 (3.4%)	82 (1.0%)	79 (1.0%)	125 (1.6%)	**3,157 (44.8%)**	28 (0.6%)	26 (0.6%)	**7,043**
Oral diseases	677 (0.7%)	286 (0.8%)	71 (0.5%)	58 (0.5%)	53 (0.6%)	92 (1.1%)	53 (0.7%)	28 (0.4%)	**3,686 (76.3%)**	5 (0.1%)	**4,832**
Injuries/Trauma	426 (0.4%)	191 (0.5%)	72 (0.5%)	71 (0.6%)	19 (0.2%)	18 (0.2%)	85 (1.1%)	26 (0.4%)	5 (0.1%)	**3,073 (73.7%)**	**4,168**
**Overall total**	**101,266**	**40,669**	**13,892**	**11,519**	**8,335**	**8,137**	**8,028**	**7,043**	**4,832**	**4,168**	**209,734**

The bold figures across the table diagonal show mono diagnoses.

The percentages in the upper section of the table diagonal are column percentages and are calculated by dividing the number of patients (N) by the column total.

The percentages in the lower section of the table diagonal are row percentages and are calculated by dividing the number of patients (N) by the row total.

The percentages in the table diagonal (mono diagnoses) are calculated by dividing the number of patients (N) by either row or column total. Column and row totals for these table diagonal are equal.

## Discussion

This study, which included 209,734 visits across 36 health facilities, presents a panoramic view of primary care provided in Uganda. Infants and children under five appeared to have equal access to care, regardless of gender, in contrast to some societies where males are disproportionately represented in the under-five population receiving care [35]. In the population 14–49 years, women predominated, with ratio of over 2:1, possibly reflecting men’s reluctance to seek care.

The disease prevalence pattern found in this study matches national patterns where data is available, with malaria (48.3%), cough or cold (19.4%), and intestinal worms (6.6%) being the most prevalent illnesses [17]. The observed malaria prevalence (47.2%) among those with confirmed malaria was also comparable to the community parasitemia prevalence reported in the 2009 Uganda malaria indicator survey [36]. The patterns of commonly reported illnesses found in this study are also similar to those observed in most SSA countries outside of Uganda [1]. This study also provides the prevalence of less common infections that are rarely reported.

Although 1,189 (0.5%) infections of epidemic potential were identified during this period, little is known about investigations done to confirm these diagnoses or control measures instituted. Reports from primary healthcare providers are essential for the early recognition and control of reportable infectious diseases that may place populations at high risk of life threatening epidemics [37]. Ebola outbreaks have occurred on four occasions during the past decade in Uganda and early recognition limited the epidemics. Rapid analysis of HMIS data could identify epidemics early to curtail and control these outbreaks [37–39].

At 10.9% of total diagnoses, NCD and injuries in Uganda are increasingly becoming an important public health concern, as in the rest of SSA countries [1–7]. Stroke (600–2400 DALYs lost per 100,000) and heart disease (300–600 DALYs lost per 100,000) are rapidly increasing causes of death and disability in SSA, with much of this tragic outcome possibly due to longstanding, untreated hypertension [2, 6, 40–42]. While diagnosis and treatment of hypertension are cost-effective and feasible within primary care, care for chronic illnesses in general requires radically different strategies, including an emphasis on patient education to facilitate treatment adherence, retention in care, and periodic tests of care effectiveness. Given that only 2.6% of the visits were follow-up for ongoing illnesses, and 0.8% of the patients had their BP recorded, further efforts and strategies will be needed to build capacity to care for chronic diseases and health outcomes generally [43].

The prevalence of multiple diagnoses or comorbidities among patients diagnosed with malaria, respiratory infections and diarrhea was high and comparable to other recent studies from SSA [1]. In our study, 30.2% of all patients and 38.9% of patients less than five years had multiple diagnoses, which is higher than findings from a Tanzania study [44] where it was 22.6% among young outpatients. Overlap in diagnoses was commonest in patients with malaria, cough/cold, pneumonia, diarrhea and intestinal infections. Of those diagnosed with malaria, our study found that 21.9% were also diagnosed with cough/cold and 5.2% with pneumonia, compared to respectively 29.5% and 7.6% in Tanzania [44]. This rate of malaria and pneumonia co-infection in young children is much lower than the 37% reported in a study done 10 years ago in Uganda [45]. In western Kenya [46], 28% of children with respiratory viral infections had malaria, which is much lower than in 61.4% of children with cough/cold in our study. These data illustrate the difficulties in making a differential diagnosis between malaria, pneumonia and even a gastrointestinal infection in situations when recommended clinical and laboratory assessments are not routinely performed. Few studies have however examined comorbidities or multiple diagnoses among outpatient populations in SSA [21, 45, 47].

In order to make these diagnoses, it is expected that clinicians will take a patient’s history and conduct basic physical assessment. Clinical history was recorded only 57.6% of the time and limited use of clinical assessment tools was observed. Weight was recorded in only 38.8% of patients more than two years old, 53.8% of patients aged one month to two years, and 38.1% of neonates. Only 2.1% of adults aged 14 or more years had a BP recorded. Proper clinical assessments are critical to ensuring high quality of care, but are rarely conducted in SSA. In an era where NCD prevalence in SSA is increasing [1–7], failure to screen for BP [48] and body mass index is a missed opportunity in assessing a patients’ potential risk.

Despite laboratory confirmations being one of the most important mechanisms for clinicians to provide more accurate diagnoses, laboratory diagnostics were rarely used, with less than 30% of patients receiving laboratory confirmation for diagnoses requiring an investigation. All sites had trained laboratory technicians and a functional laboratory that could conduct at least six investigations (HIV rapid tests, malaria blood smears, TB sputum smears, urinalysis, stool analysis, and hemoglobin estimation) [30]. When the laboratory investigations were ordered results were recorded only 50.0% to 91.4% of the time. These rates of recording differed by laboratory test with malaria test results most often recorded and sickle cells test least often. Laboratory personnel may prioritize tests that are faster to conduct or those that are more common and can easily be done in batches. At the same time, patients may be less inclined to wait for results that take longer to be recorded. As such, tests that are carried out more quickly are more likely be recorded on the MF5 and returned to the clinician for a diagnosis.

Even when results were recorded, clinicians often appeared to lack confidence in the results. Apart from malaria (79.1%), many patients with a positive test result did not have a record of a diagnosis for a related illness. Further investigations are needed to determine reasons for this difference. It is also clear many diagnoses that required a laboratory confirmation were made without them given that only 28.8% of visits for whom a laboratory investigation was needed and available to confirm the clinical impression had the specific laboratory test performed. This underuse of laboratory diagnostics may be leading to many missed or incorrect diagnoses [49, 50].

Strengthening of laboratory resources has been previously identified as an important priority in improving health services [24]. Increasing the use of laboratory services requires an appreciation of their value, as well as confidence in their reports [51, 52]. Between 2006 and 2012, IDI implemented two projects, Joint Uganda Malaria Training Program and IDCAP, in which they trained clinicians, nurses and laboratory personnel in optimal management of malaria and other infectious illnesses. The training also encouraged shared problem solving and appreciation for each other’s contribution to managing the patient. Results showed improved skills, efficient use of laboratory resources and reduction in both costly overtreatment and potentially life threatening inappropriate treatment of malaria patients [28, 51, 52].

Large databases collected by hundreds of busy individuals must be interpreted with caution. Although missing information, potential erroneous diagnoses [21, 23, 24], ability to classify diseases according to the ICD-10 which was limited by lack of specificity of diagnosis recorded on the MF5, errors in transcription and inadequacies of the MF5 instrument itself can be considered a potential limitation of our study, this large database of outpatient visits provides a unique opportunity to better understand routine care practices, particularly in rural areas.

## Conclusion

This analysis provides evidence that there is ample room for improvement in primary care, both for the treatment of infectious and, increasing NCD in rural Uganda. A strong national HMIS has the potential to enable timely collection of high quality BOD data that improves disease surveillance, curtails outbreaks and informs development of national guidelines, programs, and policies that are cost-effective, efficient and focused on local conditions. Such information could then be used to ensure accountability in the health sector and leverage improvements in the quality of care.

## Electronic supplementary material

Additional file 1: Table S1: Distribution of all diagnoses made at primary care health facilities. This file has a list of all the diseases that were diagnosed during the study period. (XLSX 35 KB)

Additional file 2: Table S2: Distribution of commonly diagnosed diseases also categorized by age groups. This table shows the analysis and distribution of the commonly diagnosed illnesses by category. (DOCX 167 KB)
