# Supplementary material for: Disease diagnosis in primary care in Uganda
Source: BMC Fam Pract. 2014 Oct 8;15:165. doi: 10.1186/1471-2296-15-165 (PMC4288627; doi:10.1186/1471-2296-15-165)
Supplement: Supplementary file 2 — Additional file 2: Table S2: Distribution of commonly diagnosed diseases also categorized by age groups. This table shows the analysis and distribution of the commonly diagnosed illnesses by category. (DOCX 167 KB) [file 12875_2014_1139_MOESM2_ESM.docx]

**Supplementary Table S2. . Distribution of commonly diagnosed diseases also categorized by age groups**

| **No** | **Diagnosis** | **National (MoH – HMIS reported)†** | **N = 209,734** | | **Mean age** | **Median age** | **Sex Ratio F/M** | **Laboratory confirmed N (%)** | **Multiple diagnoses****  **N (%)** | **Percent of patients within each age category with diagnosis** | | | | | |
| --- | --- | --- | --- | --- | --- | --- | --- | --- | --- | --- | --- | --- | --- | --- | --- |
|  |  |  | **N** | **% (95%CI)** |  |  |  |  |  | **< 1mth (n=700)** | **1-11mth (n=18,014)** | **1-4 years (n=41,978)** | **5-13 years (n=35,572)** | **14-49 years (n=94,743)** | **50+ years (n=18,727)** |
|  | **Top 20 diagnoses** |  |  |  |  |  |  |  |  |  |  |  |  |  |  |
| 1 | Malaria | 47.3 | 101,266 | 48.3 (48.1, 48.5) | 16.0 | 10 | 1.47 | 18,051 (17.8) | 48,954 (48.3) | 22.3 | 59.7 | 67.2 | 53.4 | 38.9 | 33.8 |
| 2 | Cough or cold | 23.7 | 40,669 | 19.4 (19.2, 19.6) | 15.0 | 9 | 1.48 | N/A | 27,295 (67.1) | 21.7 | 28.4 | 25.0 | 24.2 | 14.7 | 12.6 |
| 3 | Intestinal worms | 6.4 | 13,892 | 6.6 (6.5, 6.7) | 19.2 | 13 | 1.61 | 198 (1.4) | 10,525 (75.8) | 0.71 | 1.63 | 7.0 | 10.5 | 6.1 | 6.2 |
| 4 | GI disorders* | 2.8 | 11,519 | 5.5 (5.4, 5.6) | 31.6 | 30 | 2.32 | N/A | 6,347 (55.1) | 3.00 | 1.88 | 1.33 | 2.26 | 8.11 | 11.3 |
| 5 | Pneumonia | 3.2 | 8,335 | 3.9 (3.9, 4.1) | 12.4 | 3 | 1.21 | N/A | 6,003 (72.0) | 9.14 | 9.86 | 6.78 | 3.13 | 2.12 | 2.82 |
| 6 | Diarrhea | 3.6 | 8,137 | 3.9 (3.8, 4.0) | 9.5 | 1 | 1.15 | N/A | 6,409 (78.8) | 4.57 | 13.04 | 6.84 | 2.54 | 1.74 | 1.8 |
| 7 | Skin diseases | 3.7 | 8,028 | 3.8 (3.7, 3.9) | 17.0 | 12 | 1.26 | N/A | 3,689 (46.0) | 7.43 | 3.74 | 4.23 | 5.25 | 3.22 | 3.2 |
| 8 | Urinary Tract Infection | 2.3 | 7,043 | 3.4 (3.3, 3.4) | 28.4 | 27 | 1.99 | 500 (7.1) | 3,886 (55.2) | 5.14 | 2.74 | 1.11 | 0.73 | 5.10 | 5.1 |
| 9 | Oral diseases | _ | 4,832 | 2.3 (2.2, 2.4) | 24.6 | 21 | 1.48 | N/A | 1,146 (23.7) | 2.71 | 1.64 | 1.24 | 2.22 | 2.69 | 3.5 |
| 10 | Injuries, Trauma* | _ | 4,618 | 2.2 (2.1, 2.3) | 25.5 | 22 | 0.90 | N/A | 1,095 (23.7) | 0.71 | 0.48 | 0.87 | 2.61 | 2.84 | 2.9 |
| 11 | Eye conditions | 2.4 | 3,782 | 1.8 (1.7, 1.9) | 21.5 | 16 | 1.20 | N/A | 1,736 (45.9) | 3.71 | 2.12 | 1.59 | 1.75 | 1.68 | 2.64 |
| 12 | Ear Nose & Throat Conditions | _ | 3,762 | 1.8 (1.7, 1.9) | 21.1 | 17 | 1.62 | N/A | 1,787 (47.5) | 0.57 | 1.13 | 1.35 | 2.36 | 1.91 | 1.82 |
| 13 | Pelvic Inflammatory Disease | _ | 3,486 | 1.7 (1.6, 1.7) | 32.0 | 30 | N/A | N/A | 1,790 (51.4) | N/A | N/A | N/A | N/A | 3.36 | 1.61 |
| 14 | Sexually Transmitted Infections | 2.8 | 3,338 | 1.6 (1.5, 1.7) | 30.9 | 28 | 2.19 | 117 (3.5) | 1,491 (44.6) | 0.00 | 0.00 | 0.00 | 0.00 | 3.22 | 1.6 |
| 15 | Anemia* | _ | 1,515 | 0.72 (0.69, 0.76) | 10.3 | 2 | 1.19 | 408 (27.0) | 1,307 (86.3) | 0.86 | 1.95 | 1.62 | 0.36 | 0.28 | 0.47 |
| 16 | Cardiovascular – High Blood Pressure* | _ | 1,437 | 0.69 (0.65, 0.73) | 52.6 | 55 | 2.76 | N/A | 748 (52.1) | 0.00 | 0.02 | 0.03 | 0.07 | 0.52 | 4.83 |
| 17 | Typhoid fever | _ | 1,390 | 0.66 (0.63, 0.70) | 28.5 | 25 | 1.52 | 302 (21.7) | 829 (59.6) | 0.00 | 0.07 | 0.14 | 0.46 | 1.03 | 0.93 |
| 18 | HIV/AIDS | _ | 1,275 | 0.61 (0.58, 0.64) | 31.2 | 32 | 1.82 | 251 (19.7) | 720 (56.5) | 0.00 | 0.12 | 0.20 | 0.21 | 1.00 | 0.78 |
| 19 | Asthma* | _ | 1,095 | 0.52 (0.49, 0.55) | 36.5 | 36 | 1.58 | N/A | 475 (43.4) | 0.00 | 0.06 | 0.15 | 0.42 | 0.55 | 1.88 |
| 20 | Arthritis, all cases | _ | 1,074 | 0.51 (0.48, 0.54) | 44.3 | 46 | 2.43 | N/A | 453 (42.2) | 0.00 | 0.04 | 0.04 | 0.16 | 0.56 | 2.48 |
|  | **Top 5 diseases of epidemic potential** |  |  |  |  |  |  |  |  |  |  |  |  |  |  |
| 1 | Dysentery | 0.37 | 915 | 0.44 (0.41, 0.47) | 18.4 | 14 | 1.24 | 41 (4.5) | 558 (61.0) | 0.29 | 0.51 | 0.60 | 0.30 | 0.41 | 0.37 |
| 2 | Acute Flaccid paralysis | 0.00 | 77 | 0.04 (0.03, 0.05) | 22.3 | 15 | 2.67 | N/A | 60 (77.9) | 0.14 | 0.02 | 0.04 | 0.04 | 0.03 | 0.06 |
| 3 | Meningitis (Meningococcal) | 0.00 | 66 | 0.03 (0.02, 0.04) | 18.7 | 13.5 | 0.89 | 0 (0) | 23 (34.9) | 0.00 | 0.03 | 0.02 | 0.05 | 0.03 | 0.02 |
| 4 | Measles | 0.00 | 45 | 0.02 (0.015, 0.03) | 6.3 | 2 | 0.8 | N/A | 33 (73.3) | 0.00 | 0.06 | 0.05 | 0.02 | 0.00 | 0.01 |
| 5 | Yellow Fever | 0.00 | 17 | 0.01 (0.004, 0.01) | 19 | 16 | 1.83 | N/A | 9 (52.9) | 0.00 | 0.02 | 0.00 | 0.01 | 0.01 | 0.01 |
|  | **Top 5 Neglected Tropical Diseases** |  |  |  |  |  |  |  |  |  |  |  |  |  |  |
| 1 | Schistosomiasis | _ | 78 | 0.04 (0.03, 0.04) | 24.2 | 18.5 | 1.33 | 4 (5.1) | 58 (74.4) | 0.00 | 0.02 | 0.04 | 0.03 | 0.04 | 0.06 |
| 2 | Leprosy | _ | 45 | 0.021 (0.02, 0.03) | 33.0 | 40 | 1.00 | 0 (0) | 32 (71.1) | 0.00 | 0.01 | 0.01 | 0.01 | 0.02 | 0.05 |
| 3 | Filiarisis | _ | 44 | 0.021 (0.01, 0.03) | 40.5 | 40 | 2.39 | 0 (0) | 27 (61.4) | 0.00 | 0.00 | 0.00 | 001 | 0.03 | 0.05 |
| 4 | Onchocerciasis | _ | 31 | 0.015 (0.01, 0.02) | 27.5 | 25 | 2.88 | 1 (3.2) | 17 (54.8) | 0.00 | 0.01 | 0.01 | 0.01 | 0.02 | 0.02 |
| 5 | Guinea worms | _ | 19 | 0.009 (0.001, 0.01) | 18.1 | 11 | 1.71 | N/A | 13 (68.2) | 0.00 | 0.01 | 0.01 | 0.01 | 0.01 | 0.01 |
|  | **Top 5 maternal and perinatal conditions‡** |  |  |  |  |  |  |  |  |  |  |  |  |  |  |
| 1 | Abortion | _ | 298 | 0.14 (0.13, 0.16) | 25.6 | 24.0 | N/A | N/A | 89 (29.9) | N/A | N/A | N/A | N/A | 0.31 | 0.01 |
| 2 | Perinatal conditions in newborns | _ | 81 | 0.04 (0.03, 0.05) | 0.00 | 0.00 | 0.98 | N/A | 18 (22.2) | 11.6 | N/A | N/A | N/A | N/A | N/A |
| 3 | Puerperal sepsis | _ | 43 | 0.02 (0.01, 0.02) | 26.4 | 25.0 | N/A | N/A | 16 (37.2) | N/A | N/A | N/A | N/A | 0.04 | 0.01 |
| 4 | Pregnancy | _ | 43 | 0.02 (0.01, 0.02) | 24.6 | 22.0 | N/A | 3 (7) | 13 (30.2) | N/A | N/A | N/A | N/A | 0.02 | 0.01 |
| 5 | Ovarian cyst | _ | 35 | 0.02 (0.01, 0.02) | 32.5 | 30.0 | N/A | N/A | 18 (51.4) | N/A | N/A | N/A | N/A | 0.04 | 0.01 |

* Top 5 Non-Communicable Diseases

** Percent is the proportion of those with any given diagnosis who also have at least one other diagnosis

† National MOH statistics are not available for all diagnoses

**‡**Data only represents maternal and perinatal conditions that presented in the outpatient department; most patients with these conditions present at the specialized maternal and child health clinics including, antenatal, maternity and postnatal clinics.
